# The Moderating Effect of Perceived Susceptibility and Decision Conflict on the Relationship Between Comorbidities and Quality of Life Among Caregivers of People with Dementia

**DOI:** 10.1002/alz70858_100155

**Published:** 2025-12-25

**Authors:** Kyung Ah Cho, Jin Shil Kim

**Affiliations:** ^1^ Gachon University College of Nursing, Incheon, Incheon, Korea, Republic of (South)

## Abstract

**Background:**

Caregivers of people with dementia often experience compromised quality of life due to the physical and emotional burdens associated with caregiving, which can be further exacerbated by the presence of comorbidities. In this regard, perceived susceptibility and decision conflict, key psychological factors in advance care planning, may play a critical role in influencing the relationship between comorbidities and quality of life. This study aims to explore the moderating roles of perceived susceptibility and decision conflict in the relationship between comorbidities and quality of life among caregivers of people with dementia, while controlling for covariates such as age, education, and depressive symptoms.

**Method:**

In this cross‐sectional, correlational study, data on comorbidities, quality of life, perceived susceptibility, decision conflict, and sociodemographic and clinical covariates were collected from 92 caregivers of people with dementia. The moderation effects were analyzed using the PROCESS macro for SPSS (Model 2).

**Result:**

Comorbidities were significantly negatively associated with quality of life (β = ‐0.016, *p* < .001). Decision conflict (β = ‐0.001, *p* < .001) and perceived susceptibility (β = 0.004, *p* < .001) significantly moderated this relationship (F = 14.39, *p* < .001, R^2^ = .581). Conditional effects analysis revealed that the negative association between comorbidities and quality of life was strongest among caregivers with high decision conflict and low perceived susceptibility. In contrast, caregivers with lower decision conflict and higher perceived susceptibility showed attenuated or reversed effects of comorbidities on quality of life.

**Conclusion:**

This study on the quality of life of caregivers of people with dementia confirmed that perceived susceptibility and decision conflict are critical psychological factors moderating the negative impact of comorbidities. Interventions aimed at enhancing perceived susceptibility and alleviating decision conflict may effectively improve caregivers' quality of life. These findings provide a practical foundation for developing interventions to support caregivers.

